# Impact of the crystal orientation of Fe-doped lithium niobate on photo-assisted proton exchange and chemical etching

**DOI:** 10.1038/s41598-017-16454-7

**Published:** 2017-12-01

**Authors:** Shaobei Li, Guohong Liang, Zhitao Zan, Lihong Shi, Wenbo Yan, Chao Liang, Feifei Li, Lipin Chen, Bolin Fan, Xuliang Wang, Xuju Jiang, Hongjian Chen

**Affiliations:** 1 School of Materials Science and Engineering, Hebei Engineering Laboratory of Photoelectronic Functional Crystals, Hebei University of Technology, Tianjin, 300130 China; 2 School of Chemical Engineering and Technology, Tianjin University, Tianjin, 300072 China; 3 Tianjin Urban Construction Institute, Tianjin, 300384 China; 4 Bruker (Beijing) Scientific Technology, Beijing, 100081 China

## Abstract

Photo-assisted proton-exchange (PAPE) is carried out on the +c- and y-surfaces of Fe-doped LiNbO_3_ crystals and the impact of the crystal orientation on the PAPE and the subsequent photo-assisted chemical etching (PACE) is investigated. The proton distributions and the morphologies of the proton-exchanged surfaces are studied by using Micro-FT-IR, Micro-Raman, optical and scanning electron microscopy. Through the PAPE process the proton-exchange can be confined in a specific region by an incident laser beam with fixed intensity profile. It is found that the y-surface is much more fragile than the +c-surface and that micro-cracks are easily generated on the y-surface during the PAPE process. Moreover, the range and number of these micro-cracks can be controlled by the experimental parameters of the PAPE process. The etching morphology of the y-surface shows apparent directional features along the c-axis of LiNbO_3_ crystal and the proton spatial distribution is found elongated along the c-axis. Both effects are attributed to the accumulation of photovoltaic charges at the two sides of the illumination area along the c-axis.

## Introduction

Lithium niobate (LN) is a potential material for fabricating integrated optical components due to its outstanding electro-optic and nonlinear optical properties^1^. Proton exchange (PE)^2–12^ is known as a basic technology for the surface modification of LN substrate, for example, fabricating metal nanoparticles on the surface or tuning the refractive index of the surface. The conventional PE is essentially a proton incorporation in the LN surface, and it happens through a reaction of Li-H ion exchange in an acidic bath, where the bath temperature are usually set in the range of 120~300 °C for the proton activation^2,3^. For a summary of the basic knowledge about PE we recommend ref.^3^, where Cabrera *et al*. reviewed the previous important experimental results and the physical bases of the PE process in LN.

Many researchers contributed to the technique and theory of the conventional PE process. Korkishko and Fedorov *et al*.^6^ carefully studied the PE process in LN and established a correlation between the crystal phase structure and its refractive indices. Kostritskii *et al*. investigated the dependence of EO properties on the PE phase composition by varying the PE conditions^10^. Cabrera and his colleagues put efforts on the optimization of the PE procedures for waveguide fabrication^3,7–9,11,12^. For the first time they proposed the reverse proton exchange (RPE) process, which consists of performing a reverse exchange in a melt rich in lithium on a previously proton-exchanged LN substrate^11^. The waveguides fabricated by RPE have nearly-symmetrical refractive index profiles and therefore the RPE technique presents a great potential for the realization of all-optical devices^12,13^. While many scientific works analyzed the impact of the PE parameters on the subsequent light propagation through the processed crystal, it appears that almost no investigation has been carried out regarding the impact of light beams illuminating the crystals during the PE process^14^.

In this paper, we report a photo-assisted PE (PAPE) effect on Fe-doped LN (LN:Fe) crystals. Herein, the PE process is activated by laser irradiation in an acidic bath kept at 60 °C, which is not a sufficient temperature for the conventional proton activation. In other words, even below the conventional proton-activation temperature the laser irradiation still can induce the proton incorporation in the LN surface or the further chemical-etching (PACE) of the LN surface, depending on the parameters of the laser irradiation and the orientation of the LN crystals^14^. Both the circular and triangular intensity profiles of the incident laser beams are attempted and the distribution of the relative proton concentration is found well controlled. Moreover, the PAPE features for different crystal orientations are analyzed and explained. The results shown in this work may be useful for the refractive index engineering or micro-structure fabrication on the LN surface.

## Experimental procedures

**Figure 1:**
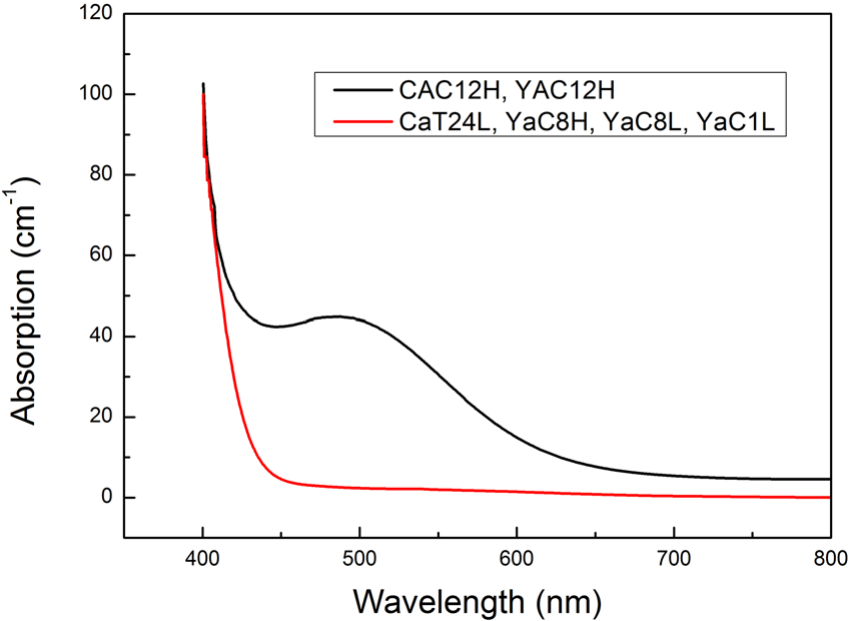
UV-VIS spectra of the Fe-doped LN samples 1~6, labeled as CAC12H, CaT24L, YAC12H, YaC8H, YaC8L and YaC1L, respectively.

The samples used in this work are 1mm-thick, Z-cut or Y-cut LN:Fe crystals, and the Fe_2_O_3_ doping concentration is 0.03 wt%. The crystals are grown at the congruent point and the composition ([Li]/[Li] + [Nb]) equals to 48.4/51.6. The samples are polished to the optical grade. In order to modify the sample absorption, we anneal the samples for 3 h in vacuum (10^−1^ Pa) at different temperatures (500 or 1000 ^o^C). Figure 1 shows the UV-VIS absorption spectra of the samples. The information of these samples is listed in Table 1. Note that an un-polarized light is used for absorption measurement and therefore the sample orientation does not modify the result too much. Since the light source used in our work is a CW laser rather than a Pulse laser, the involved intensity in the PAPE process is quite low. Thus, the light absorbed by the sample during the PAPE process is considered to be proportional with the laser intensity, and any nonlinear absorption effect can be neglected. In addition, the [Fe^2+^] of LN:Fe samples can be calculated by using the absorption at 532 nm in Fig. 1 ^15,16^. The redox state of the Fe ions, represented by the ratio [Fe^2+^]/[Fe], is listed in Table 1.

**Table 1:** PAPE experimental parameters of the different samples. To give an immediate indication of the sample properties we label the samples in the following way: use A/a for 42/4 (cm^−1^) absorption, C/T to differentiate between Circular and Triangular profiles and H/L to indicate the 400/200 (W/cm^2^) intensity.

Sample	Label	Crystal Orientation	Absorption@ 455 nm (cm^−1^)	Redox State of Fe ions [Fe^2+^]/[Fe]	Sample Annealing Temperature	Laser Beam Profile	PAPE Treatment Duration (h)	Irradiation Intensity (W/cm^2^)
1	CAC12H	+c	~42	89.1%	1000 °C	Circular	12	~400
2	CaT24L	+c	~4	5.3%	500 °C	Triangular	24	~200
3	YAC12H	y	~42	89.1%	1000 °C	Circular	12	~400
4	YaC8H	y	~4	5.3%	500 °C	Circular	8	~400
5	YaC8L	y	~4	5.3%	500 °C	Circular	8	~200
6	YaC1L	y	~4	5.3%	500 °C	Circular	1	~200

**Figure 2:**
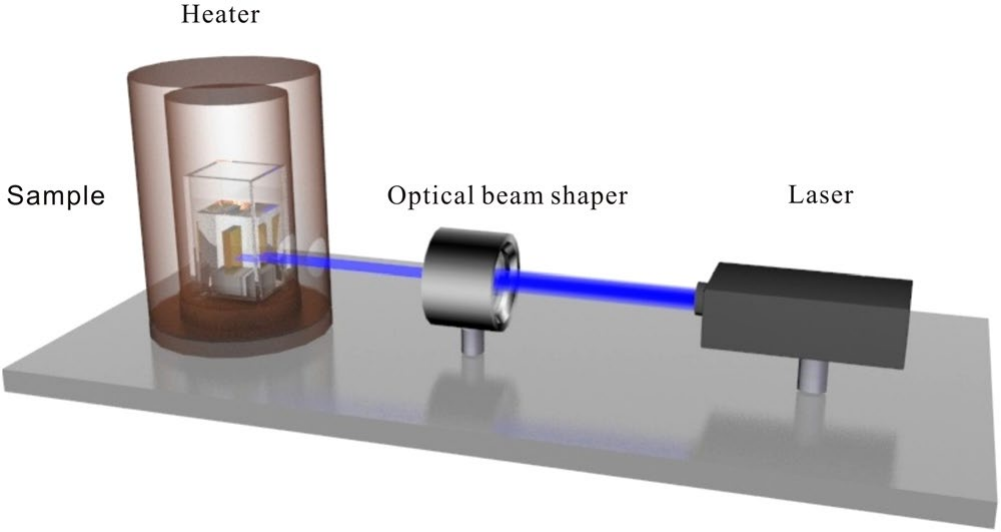
Outline of the experimental setup for PAPE treatment.

Figure 2 shows the outline of the experimental setup for the PAPE treatment. The samples are soaked in pyrophosphoric acid and subjected to laser irradiation simultaneously. The acid temperature is controlled by Eurotherm 3508 and it is set as 60 °C during the treatment. A 455-nm laser (CniLaser) beam is focused on the +c- or y-surfaces of the samples, with the laser beam profile (circular or triangular) being adjusted by an optical beam shaper in which different kinds of metal masks are installed. The basic (Gaussian) profile of laser beam is labeled here as the circular one. The triangular profile is created by modifying the basic laser beam with the optical beam shaper including a triangular-pattern mask. The diameter of the circular laser beam is measured through a knife edge experiment. Before the laser beam finally reaching the sample it has to pass a quartz beaker window and the transparent solution of the pyrophosphoric acid. However, the introduction of the beaker and the solution almost has no influence on the basic profile of the laser beam. In the experiments, the samples with different absorptions and orientations are used, and the irradiation intensity and the treatment duration are varied from one case to the other. Table 1 summarizes the experimental parameters of the different samples. To give an immediate indication of the sample properties we label the samples in the following way: use A/a for 42/4 (cm^−1^) absorption, C/T to differentiate between Circular and Triangular profiles and H/L to indicate the 400/200 (W/cm^2^) intensity.

Stand-alone FT-IR microscope (Bruker LUMOS) and Micro-Raman (Renishaw) spectrometer are used to characterize the proton distribution in the surface of the treated samples. In the IR spectrum of LN, the narrow band centered at 3500 cm^−1^ is associated with O-H stretching vibration^3,17^, and thus it is considered as the best marker of protons in crystals. For each sample, M × N mapping points are selected around the treated region and the FT-IR microscope collects point by point the IR spectrum with a resolution of 1 cm^−1^ and a scan time of 32 s/point. In order to obtain the spatial distribution of the relative proton concentration, the integration of the O-H vibration band at each point is calculated^14^ and the band area, which is proportional to the proton concentration, is plotted as a function of spatial position in a 2D way. Micro-Raman spectroscopy is a well-known tool for detecting the local variation of crystal lattice^18,19^. Therefore, we measure the Raman spectra within and without the treated region for studying the effect of PAPE on the crystal lattice. Besides, an optical microscope (Olympus STM6) and a scanning electron microscope (ZEISS MERLIN Compact) are employed to acquire the morphology of the treated surface.

## Result and Discussion

**Figure 3:**
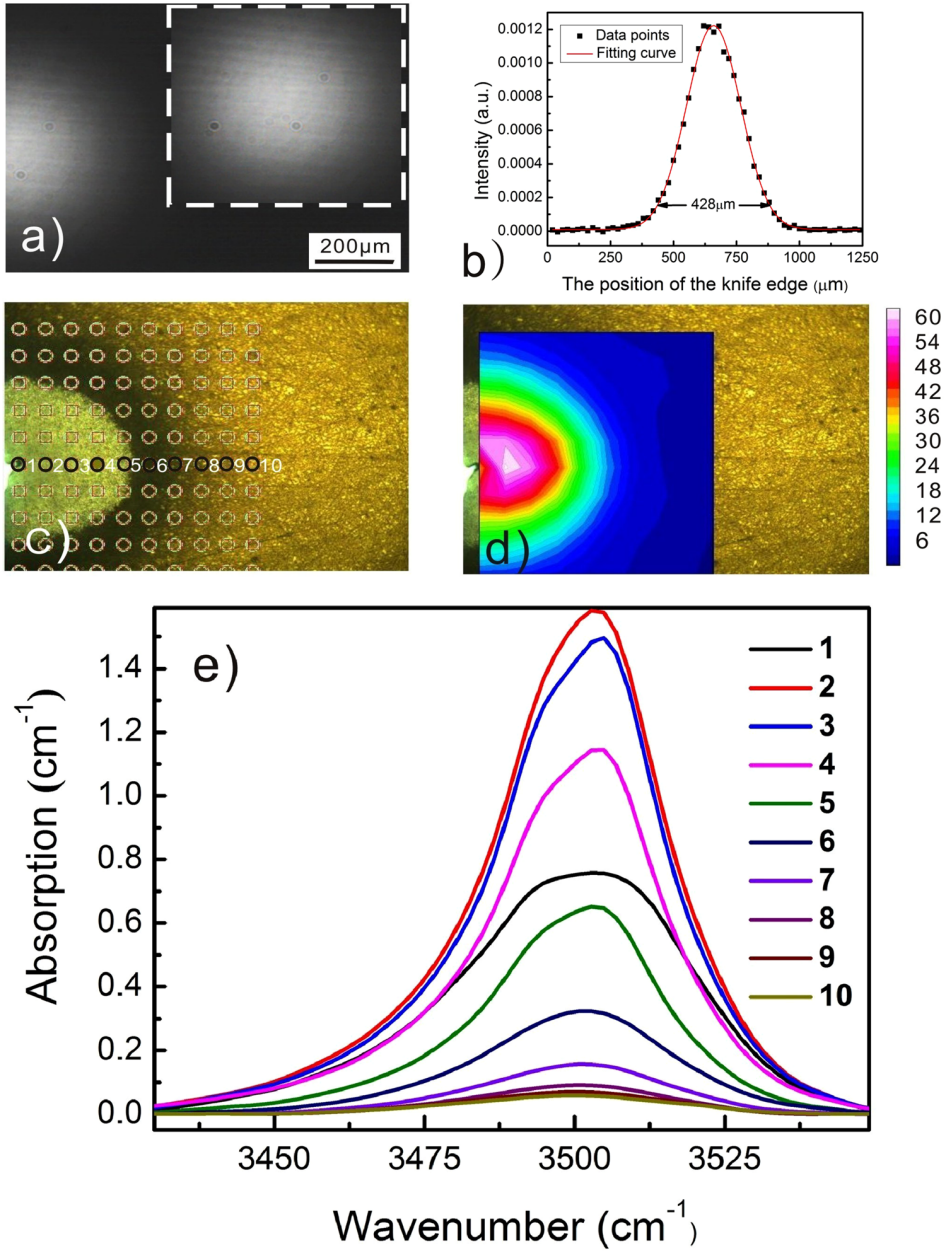
Micro-FT-IR mapping results of the sample CAC12H, in which a circular profile of the laser beam is used; (**a**) shows the non-saturated images of the circular profiles. Note that the part of profile shown here is according to the PAPE region covered by the Micro-FT-IR mapping, and the whole image of circular profile is shown in the inset; (**b**) Is the result of knife edge experiment. The diameter of the laser beam is measured to be 428 μm. (**c**) Is the lattice of the Micro-FT-IR mapping points; (**d**) is the corresponding spatial distribution of the relative proton concentration. The proton distribution follows the profile of the laser beam. (**e**) Shows the O-H vibration bands collected from the labeled mapping points.

**Figure 4:**
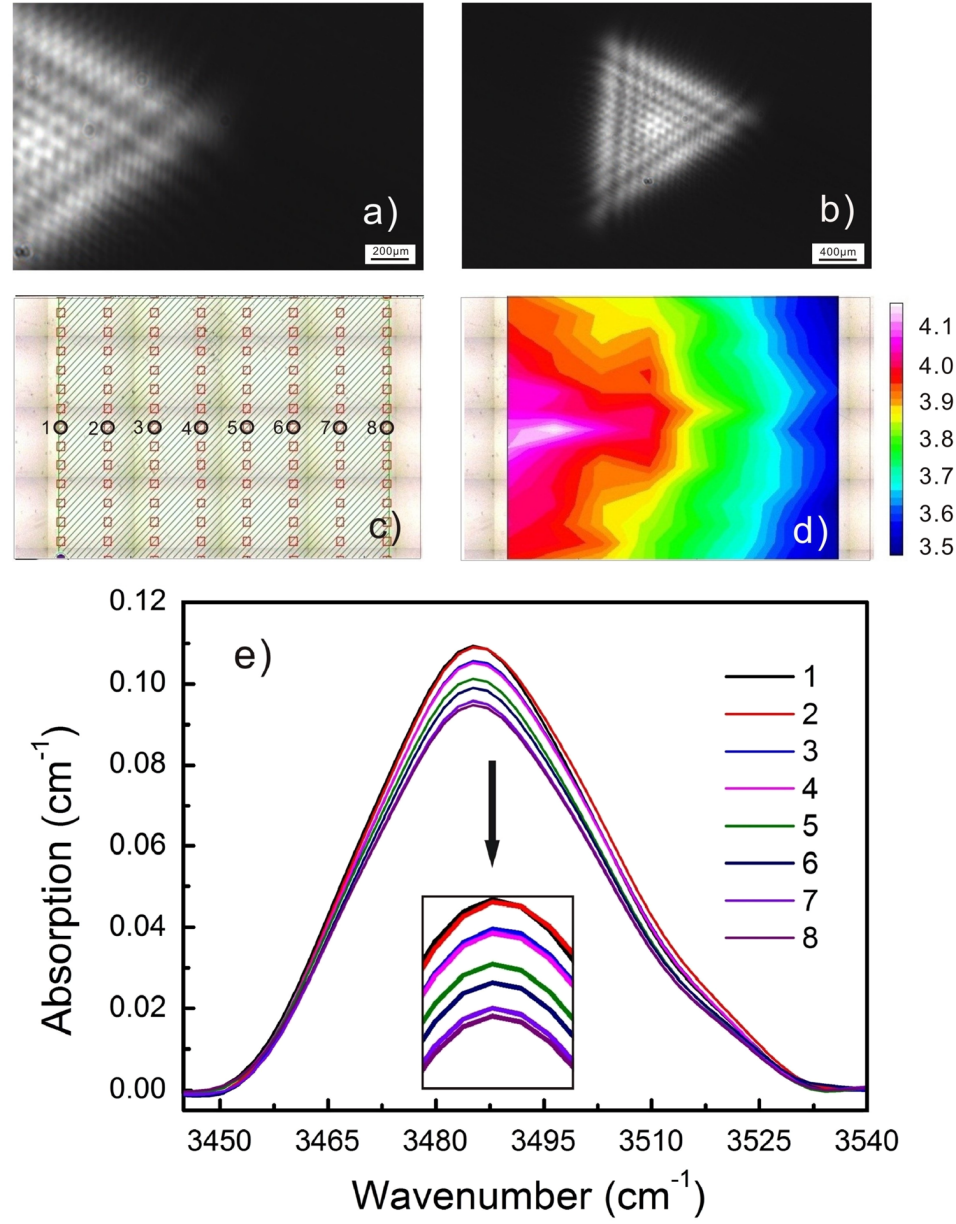
Micro-FT-IR mapping results of the sample CaT24L, in which a triangular profile of the laser beam is used; (**a**) and (**b**) are the non-saturated images of the triangular profiles. Note that the part of profile shown in (**a**) is according to the PAPE region covered by the Micro-FT-IR mapping while the whole image of triangular profile is shown in (**b**); (**c**) is the lattice of the Micro-FT-IR mapping points; (**d**) is the corresponding spatial distribution of the relative proton concentration. The proton distribution roughly follows the profile of the laser beam but its border shows a spreading trend (**e**) shows the O-H vibration bands collected from the labeled mapping points.

We perform the PAPE treatments on the +c-surface of LN by using the laser beams with circular (CAC12H) and triangle (CaT24L) profiles. Figures 3(a) and 4(a) show the laser beam profiles captured by a CCD. Figures 3(d) and 4(d) show the corresponding spatial distribution of the relative proton concentration. The lattice of the FT-IR mapping points and the O-H vibration bands collected from the labeled mapping points are given in Figs 3(c),(e) and 4(c),(e), respectively. It is found in both cases that the proton incorporation is confined in the specific region by the laser beam irradiation. In other words, the proton incorporation can be modulated by the profile of the irradiation intensity. As a matter of fact, the heavy PAPE of CAC12H causes a further PACE process while only soft proton incorporation is induced on CaT24L and no surface material removal is found.

**Figure 5:**
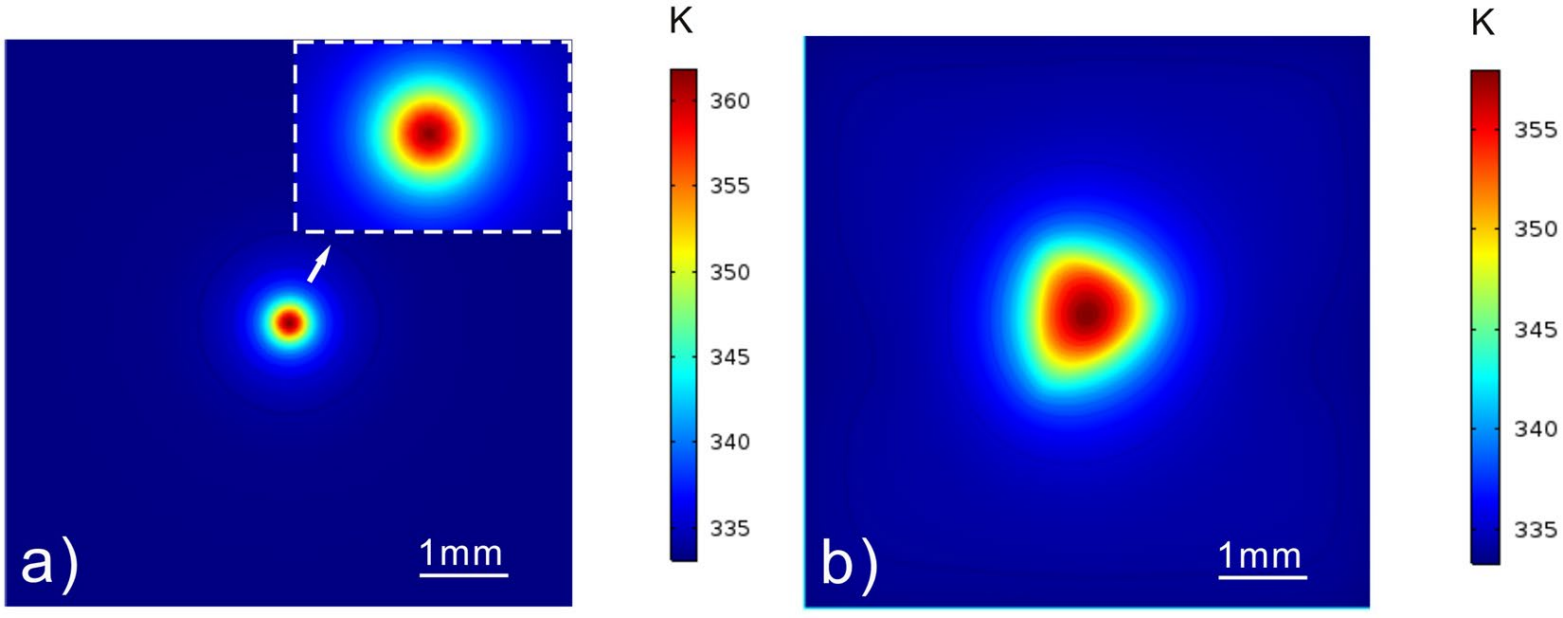
Simulation results of the temperature distribution on (**a**) CAC12H and (**b**) CaT24L. The maximum temperatures at the focus in both cases are below 100 °C, which is still not a sufficient temperature for the conventional proton activation.

The PAPE treatment using a circular profile was firstly reported in ref.^14^, where the photovoltaic effect (PVE) was considered as the main mechanism of the PAPE phenomenon. In the present work, both the circular and triangle profiles are used for the PAPE treatment. As compared with the circular profile (i.e. the Gaussian profile), the triangular profile created here is more marginated due to the mask modification to the laser beam. Moreover, the triangular profile includes many fine diffraction patterns caused by the mask edge. However, we cannot find in Fig. 4(d) any fine pattern of the proton distribution. Furthermore, the general proton distribution is quite broad and its border shows a spreading tendency. These results suggest that a thermal effect contributes partly to the PAPE effect. By using a finite-element model, we simulate the temperature distribution when irradiating the c-cut samples with the circular and triangular profiles. In the simulation, the heat induced by the light absorption is treated as an independent thermal source in a specific region. Thermal conduction is considered as the main heat-transfer mechanism inside the sample while thermal convection is considered as the main one at the boundary between the sample and the surrounding acid. The direct heat exchange between the sample and acid significantly lowers the local temperature around the irradiated region. The majority of heat dissipates through the convention into the acid. Figure 5 shows the simulation results of the temperature distribution on CAC12H and CaT24L. It can be seen that the maximum temperatures at the focus in both cases are below 100 °C, which is still not a sufficient temperature for the conventional proton activation. However, combined with the electrostatic interaction, this thermal effect may contribute partly to the proton distribution. As one can see, the temperature profile on CaT24L has no fine pattern and clear boarder, which results in the proton distribution shown in Fig. 4(d). Although the thermal effect contributes to the PAPE process, the following results on the y-cut samples demonstrate that the photovoltaic effect does play an important role in the PAPE process.

**Figure 6:**
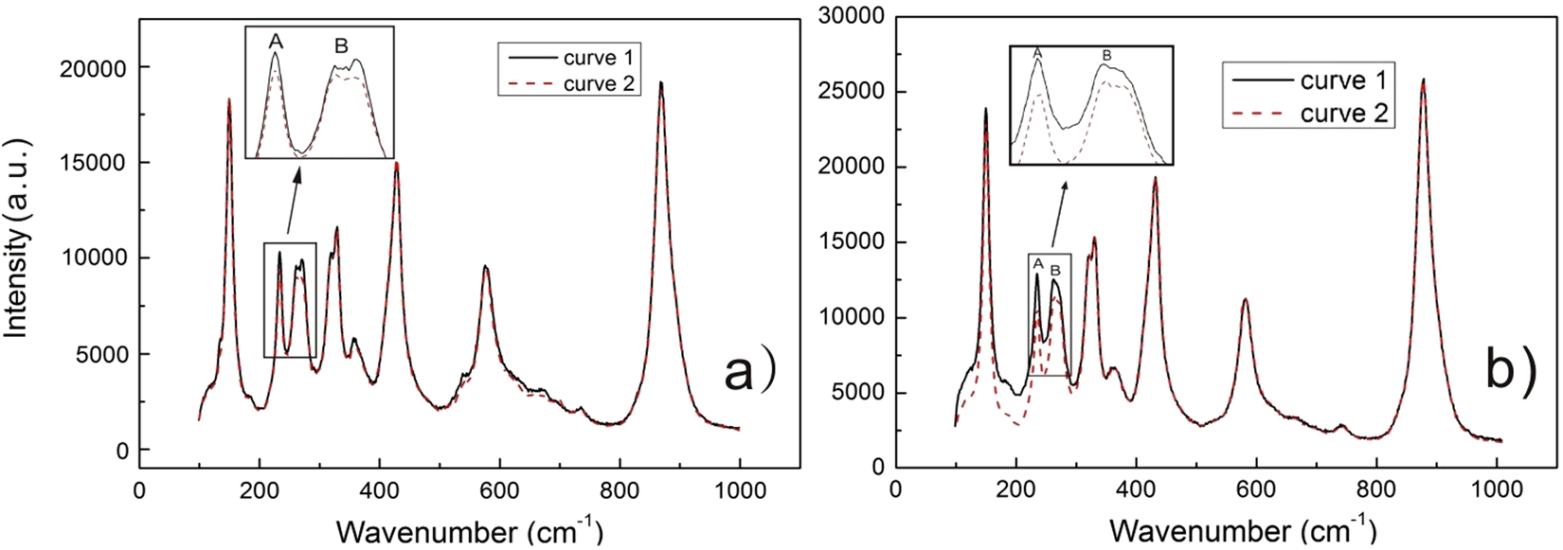
(**a**) The Micro-Raman spectra of the PAPE surface of CaT24L. Curve 2 and 1 are corresponding to the spectra collected within and without the irradiated region, respectively. As compared with the curve 1, the slight intensity reduction of Raman lines in the low wavenumber range of 100–300 cm^−1^ can be found in the curve 2. (**b**) The Micro-Raman spectra of the PAPE surface in another case, where the sample absorption is about 18 cm^−1^ and the irradiation intensity is about 400W/cm^−1^. The slight intensity reduction of Raman lines in the similar wavenumber range is also found in this case, showing a good reproducibility of our results.

Although no etching sign is found in the treated region of CaT24L, the crystal lattice still has some changes. Figure 6(a) shows the Micro-Raman spectra of the PAPE surface of CaT24L, and the two curves are corresponding to the spectra collected within and without the irradiated region, respectively. Note that the PE process happens only within the irradiated region while the protons in the dark cannot incorporate into the crystal due to the low local temperature. Generally, the proton incorporation in the PE region may induce the distortion of the local crystal lattice, leading to the intensity reduction of some Raman-lines^19^. As compared with the curve 1, we can find in the curve 2 the slight intensity reduction of Raman lines in the low wavenumber range of 100–300 cm^−1^. To test the reproducibility of this result, we repeat the experiment on another sample, for which the absorption is about 18 cm^−1^ and the irradiation intensity is about 400 W/cm^−1^. The slight intensity reduction of Raman lines in the similar wavenumber range is also found (See Fig. 6(b)). It has been proved that the Raman lines in the low wavenumber range are connected with the vibration modes of the Li sites while those in the higher wavenumber range are associated with the modes of the Nb sites^20^. Considering the protons first enter the Li sites during the incorporation^17^, we believe that the intensity reduction of Raman lines in the range of 100–300 cm^−1^ is due to the proton exchange in the irradiated region, i.e. the effect of PAPE. Except the reduction of peak intensity in the range of 100–300 cm^−1^, other PE-induced characteristics reported commonly are absent from our spectra. Spectra acquired from the PE region typically exhibit peak-broadening as well as the appearance of a high intensity peak at 690 cm^−1^. However, the spectra within and without the irradiated region are indistinguishable in the range of 300–1000 cm^−1^ in our case. This discrepancy is probably due to the much lower extent of proton incorporation in the PAPE than in the conventional PE process. As a matter of fact, the slight change of the O-H vibration bands in Fig. 4(e) is a good proof for the super-low extent of the proton incorporation in the surface of CaT24L. Such soft proton incorporation means the depth of the proton diffusion in the surface is quite short. We try to perform a depth-scan in the crystal for checking by Micro-Raman measurement the depth of the proton diffusion. However, we cannot observe any obvious depth dependence of the Raman spectrum. The possible reason is the spatial (depth direction) resolution of the Micro-Raman in our case is too low to detect the depth dependence of the proton distribution. In addition, the difference between curve 1 and 2 is so small that the depth dependence of the Raman spectrum is very hard to measure. For studying this depth dependence higher spatial resolution of the Micro-Raman and heavier proton incorporation in the PAPE process are required. Higher sample absorption and irradiation intensity are expected to enhance the proton diffusion at the irradiated region. However, any etching effect must be avoided because the Micro-Raman measurement, which is quite sensitive to the residual stress, may be influenced by the surface morphology of the etching region. Alternatively, second ion mass spectrometry (SIMS) technique could also be used to directly characterize the depth dependence of the proton distribution providing the irradiated region is large enough for the SIMS experiment.

**Figure 7:**
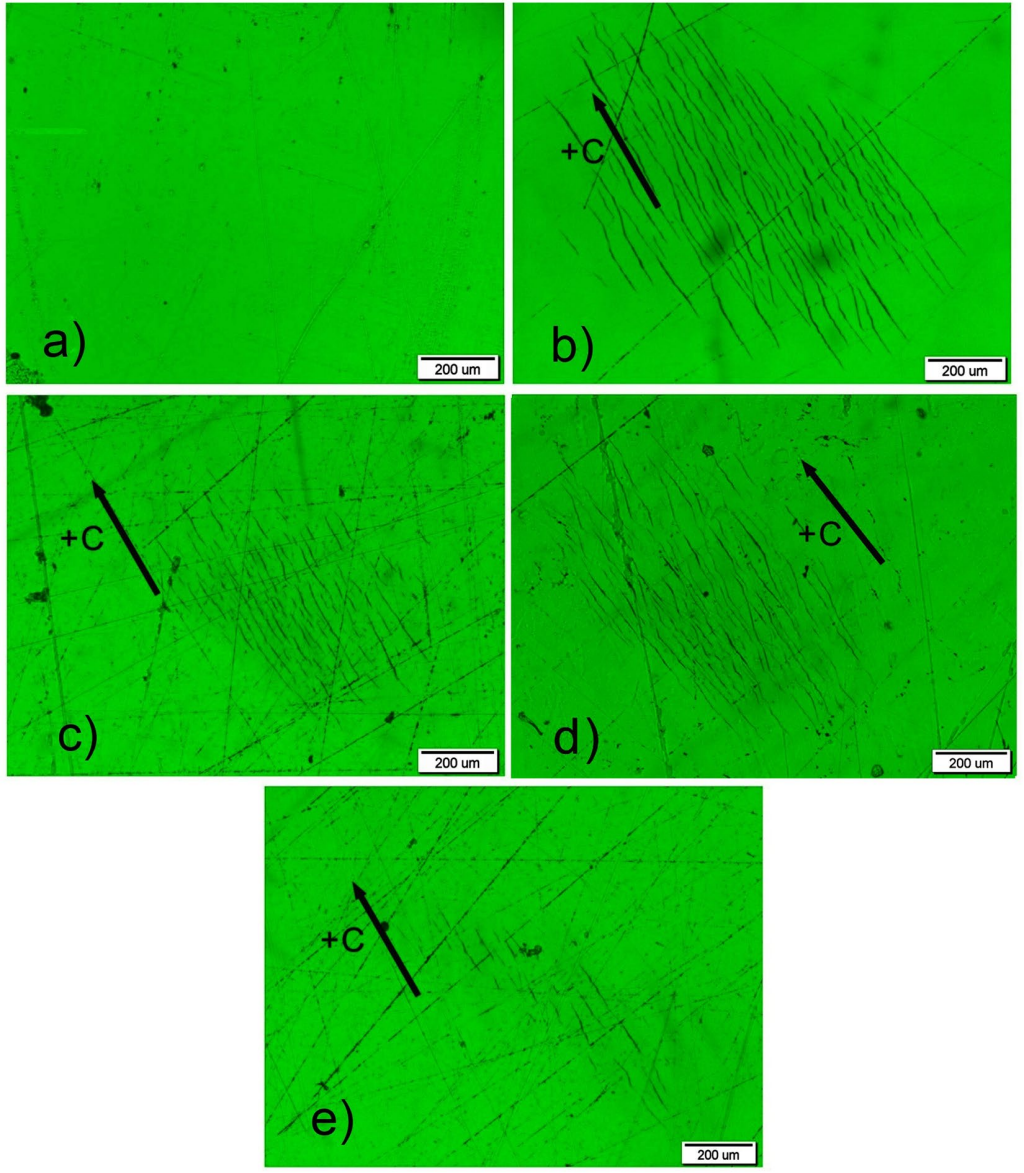
Optical microscopy images of the +c and y-surfaces of LN crystal after the PAPE treatments. (**a**) Is the image of the +c-surface of CaT24L. (**b**,**c** and **e**) are the images of the y-surface of YaC8H, YaC8L and YaC1L. The y-surface is much easier etched than the +c-surface during the treatment, and the range and number of micro-cracks can be controlled by the experimental parameters. (**d**) Is the result of the repeat experiment for YaC8L, and the similar micro-crack topography here shows a good reproducibility of our result.

**Figure 8:**
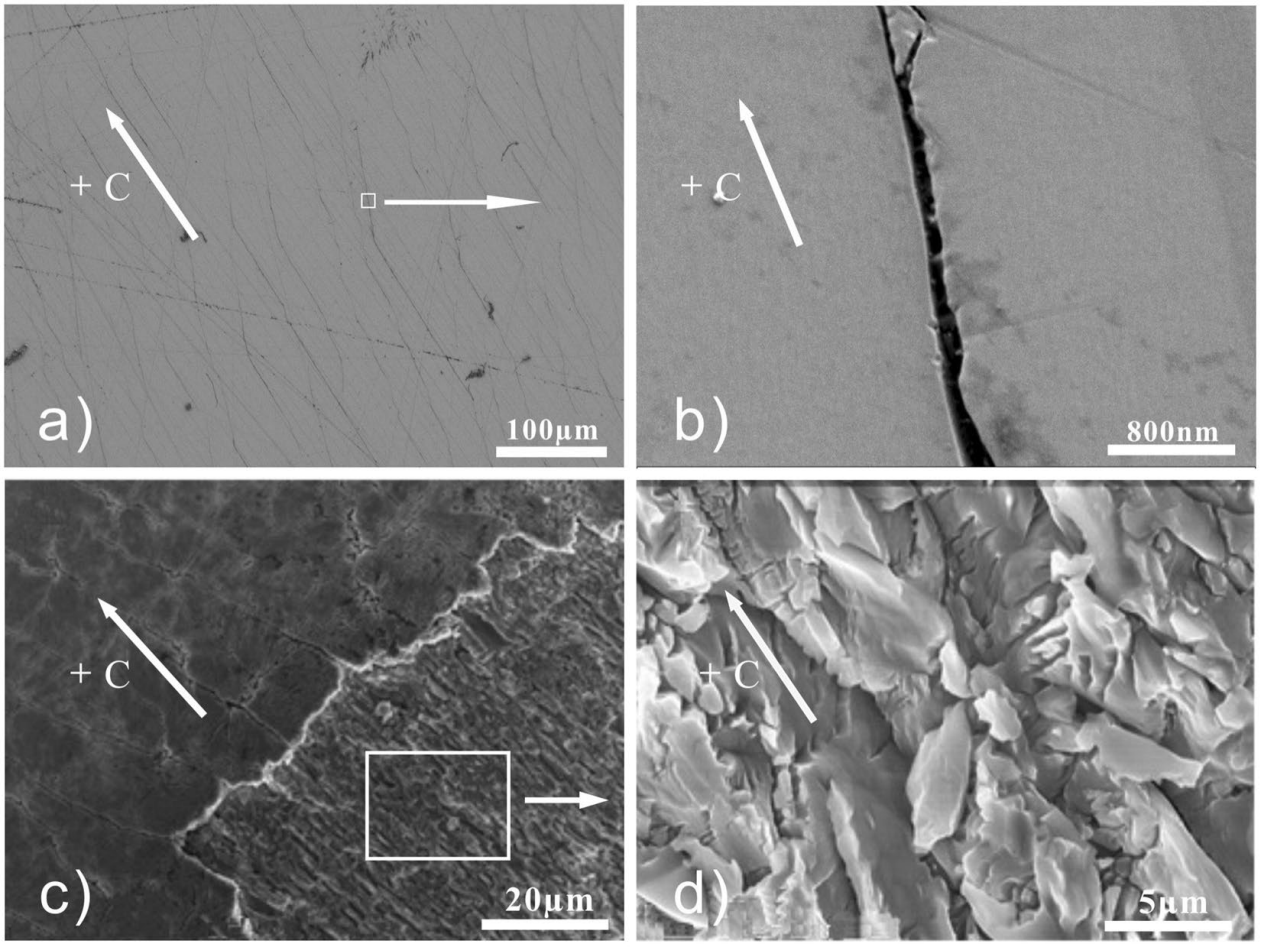
SEM images of the y-surface morphology after the PACE treatment. (**a**,**b**) and (**c**,**d**) are for YaC8H and YAC12H, respectively. (**b**) and (**d**) are the magnified images of (**a**) and (**b**).

In order to compare the PAPE results of the +c- and y-surface of LN:Fe, we carry out the experiments on the samples with the same absorption (4 cm^−1^). Figure 7(a) shows the image for the +c-surface, while 7(b), (c), (d) and (e) for the y-surface. After the PAPE treatment, the +c-surface of CaT24L remains untouched except some scratches. By contrary, a large number of micro-cracks appear on the y-surface of YaC8H, YaC8L and YaC1L. This result reveals that the +c- and y-surfaces have different resistance to the PAPE treatment, i.e. a certain extent of PAPE treatment only induces the proton incorporation on the +c-surface while it results in the chemical etching process on the y-surface. In other words, the y-surface is much more fragile than the +c-surface during the PAPE process. As a matter of fact, these micro-cracks cannot be induced alone by either the laser illumination or the acid etching, and they are a combined effect of the laser illumination and acid etching, i.e. the PACE effect. Figure 7(b),(c) and (e) show the images of the y-surface (YaC8H, YaC8L and YaC1L) under different experimental conditions (i.e. the treatment durations and irradiation intensities). It can be seen that the range and number of micro-cracks can be controlled by these experimental parameters. Figure 7(d) is the result of the repeat experiment of YaC8L, and the similar micro-crack topography here shows a good reproducibility of our result. Figure 8(a) and (b) show the magnified images of these micro-cracks acquired by SEM. The width of these micro-cracks is ranging from several tens of nanometers to few hundreds of nanometers. After a strong PACE treatment to YAC12H, parts of material can be ablated from the sample surface. Figure 8(c) and (d) show the morphology of LN surface in this case. It can be seen that the etching contrast is quite high at the boundary of the irradiated region. Moreover, the morphology of the irradiated region shows many needle-like etching structures. The magnified picture Fig. 8(d) reveals that they are lamellar etching fragments with width less than 5 µm. These etching structures originate from the initial micro-cracks on the surface. Tuning the micro-cracks and etching structures through the PACE conditions could be useful for the micro-structure fabrication on the LN surface.

**Figure 9:**
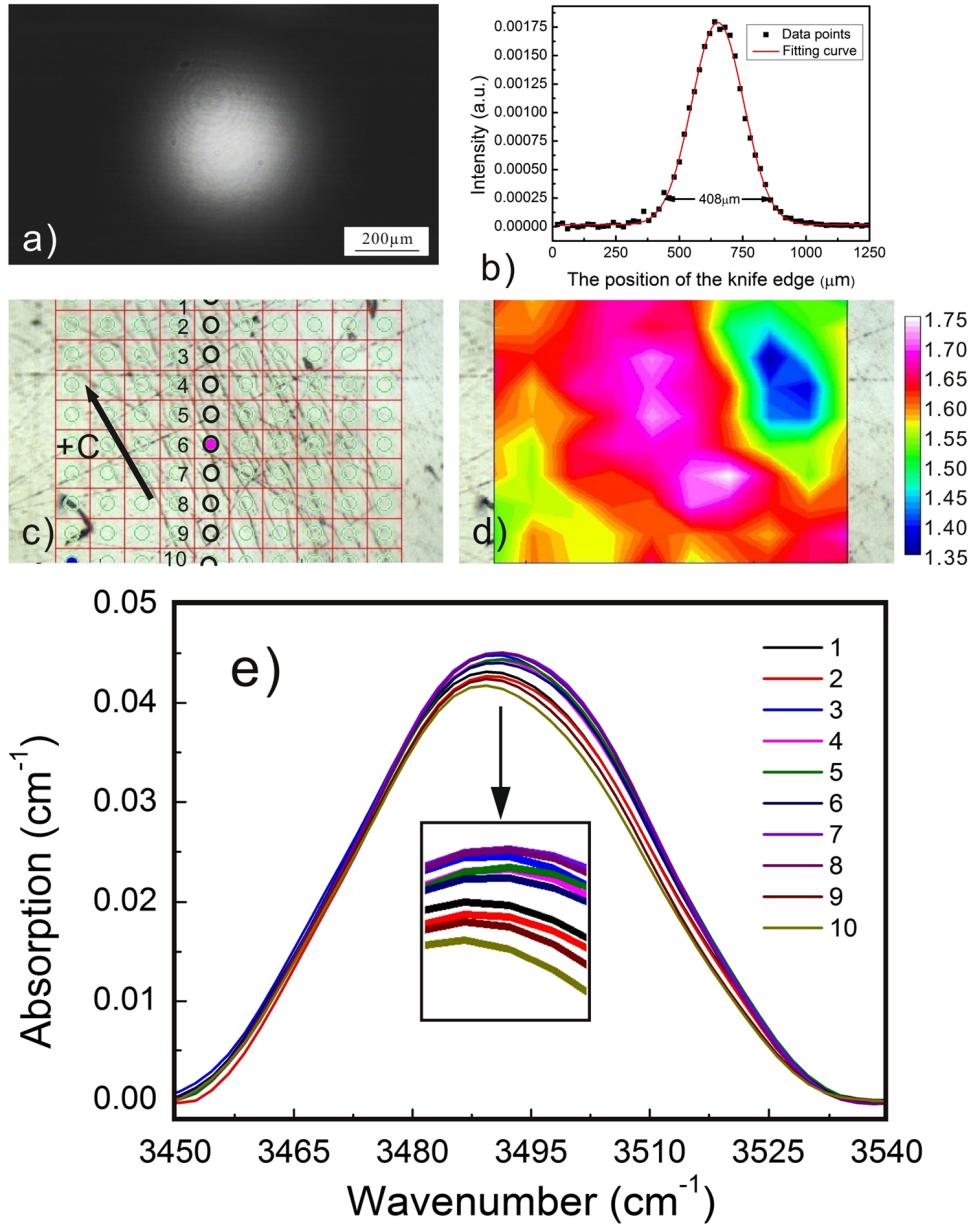
Micro-FT-IR mapping results of YaC8H, in which a circular profile is used; (**a**) is the non-saturated image of the circular profile. (**b**) Is the result of knife edge experiment. The diameter of the laser beam is measured to be 408 μm. (**c**) Is the lattice of the Micro-FT-IR mapping points; (**d**) is the corresponding spatial distribution of the relative proton concentration. The proton distribution does not entirely follow the circular profile and it seems being elongated along the c-axis. (**e**) Shows the O-H vibration bands collected from the labeled mapping points.

**Figure 10:**
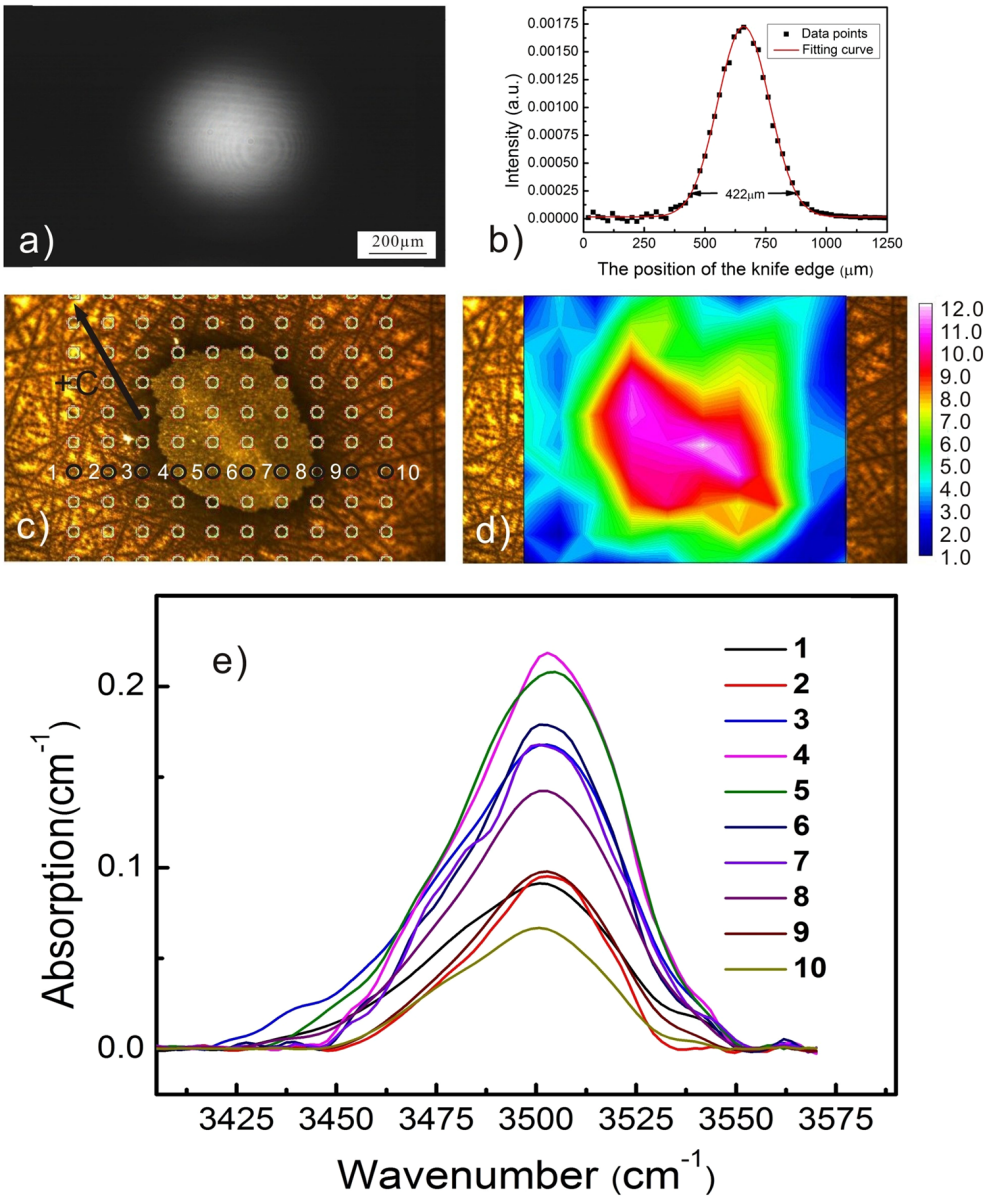
Micro-FT-IR mapping results of YAC12H, in which a circular profile is used; (**a**) is the non-saturated image of the circular profile. (**b**) Is the result of knife edge experiment. The diameter of the laser beam is measured to be 422 μm. (**c**) Is the lattice of the Micro-FT-IR mapping points; (**d**) is the corresponding spatial distribution of the relative proton concentration. The proton distribution does not entirely follow the circular profile and it seems being elongated along the c-axis. (**e**) Shows the O-H vibration bands collected from the labeled mapping points.

Another noticeable result regarding Figs 7 and 8 is that almost all of the micro-cracks are along the same direction, i.e. the c-axis of the crystal. This feature is definitely associated with the anisotropy of LN crystal. But the mechanism has to be explained basing on the proton distribution. Note that the lines that are not aligned along the c-axis in Fig. 7(b),(c) and (e) are the scratches induced by unwanted touches. We show in Fig. 9(d) that the spatial distribution of the relative proton concentration around the micro-cracks on the y-surface of YaC8H. The lattice of the FT-IR mapping points and the collected O-H vibration bands are given in Fig. 9(c) and (e). Although the laser beam profile used in Fig. 9(a) is the circular one, the proton distribution does not entirely follow the circular profile and it seems being elongated along the c-axis. We do not show here the corresponding FT-IR mapping results for YaC8L and YaC1L, because the irradiation intensities used for these samples are much lower and the proton incorporations in these cases are too soft to induce the obvious difference between the O-H vibration bands collected at mapping points. However, this feature can be also observed in Fig. 10 for YAC12H, where the proton incorporation is enhanced and parts of surface material are ablated in the PACE process. It should be noted that the band area contrast in Figs 9(e) and 10(e) are collected from the damaged surfaces. Thus, the strains induced by the micro-cracks and ablation might contribute to the band area, i.e. they may broaden the spectral bands. However, the collected O-H vibration bands in Figs 9(e) and 10(e) show an obvious increase of the band intensity after the treatment, indicating that the proton incorporation is the main origin of the band area contrast and that the directional feature of the band area contrast indeed stem from the spatially selective proton incorporation.

This anisotropy of the proton distribution and the corresponding PACE features should be connected with the photo-related anisotropy in the LN:Fe ----- photovoltaic effect. Under the irradiation, the photo-excited electrons in the crystal transport along one fixed direction, inducing a current toward the −z side. This current can lead to the accumulation of the negative and positive charges in the +z and the −z sides of the irradiated region, respectively. The separation of the positive and negative space-charges may establish a strong electrostatic field along the y-surface. Wang *et al*. reported that the electric-field distribution on LN surface may significantly influence the proton diffusion in the lateral and depth directions of the surface^21^. Thus, this photovoltaic field is highly possible to accelerate the proton exchange and the subsequent chemical etching, resulting in the anisotropy of the proton distribution and the corresponding PACE features. As a matter of fact, we also try the same treatment on pure LN and Mg-doped LN (LN:Mg) crystals, for which the visible-light-induced photorefractivity is reduced largely as compared to the LN:Fe crystals. We find no such PAPE or PACE effect in these cases. As we know, the UV-light-induced photorefractivity is quite pronounced even in pure LN and LN:Mg crystals. Thus, the focused UV laser is capable to induce such PAPE or PACE effect in these crystals. Since during the UV photorefractive process the charge transportation is governed by the diffusion field rather than the photovoltaic field, any directional features along the c-axis are expect to disappear.

As mentioned above, the proton incorporation can be modulated by the irradiation intensity profile for both c-cut and y-cut orientation. As far as the modulation depth and contrast of the proton concentration, they should be connected with several experimental parameters, i.e. the sample absorption, the irradiation intensity etc. Although we cannot provide more systematical experimental data in the present work, we can approximately consider that higher sample absorption and irradiation intensity may enhance the proton incorporation at the irradiated region and that they can increase the modulation depth and contrast of the proton concentration. This point can be supported by the two groups of comparison: (CAC12H Vs CaT24L) for the c-cut orientation and (YAC12H Vs YaC8H) for the y-cut orientation. By comparing the corresponding spatial distribution of the proton concentration in Figs 3, 4, 9 and 10, one can find that the relatively higher modulation depth and contrast of the proton concentration is obtained in CAC12H and YAC12H, where either the sample absorption or the irradiation intensity is higher than in the matched samples. To obtain the quantitative relationship between the modulation of the proton incorporation and these experimental parameters, more comparison experiments with different experimental parameters are needed. As shown by the above results, both the thermal and photovoltaic effects contribute to the PAPE process. In other words, the PAPE is essentially a process in which the protons diffuse thermally in a photo-excited electrostatic field. In order to precisely predict the proton distribution around the PAPE region, we have to establish a set of mathematic equations describing the proton transportation in a coupled field where both the thermal and electrostatic effects are taken into account. Thus, more systematical PAPE experiments and the corresponding characterizations should be carried out. For example, different irradiation intensity profiles should be attempted: irradiating the LN surface with a laser line, instead of a single spot. By varying the line direction, parallel or perpendicular to the c-axis, it would be possible to investigate the role of the E-field caused by the space-charge field. Alternatively, the laser marking system, which is capable to control the irradiation flux, can also be introduced into the PAPE setup for creating more complicated profiles. In addition, the PAPE experiments on LN:Mg crystals using UV light may bring more informative results. In practice, the LN crystals doped with photorefractive-resistant impurities usually possess a high intensity threshold against the visible optical damage, and they are the suitable substrates for waveguide fabrication. Thus, the UV-PAPE technique is quite promising for the fabrication of the photonic components on these LN crystals. The experiments mentioned above are already in our plan and the results will be published in the future.

As far as the reliability of the experimental results in the present work, we have to discuss from several aspects. Firstly, the samples used in the work are cut from the same crystal bulk and they undergo fixed processing procedures except the heat treatment conditions. Thus, the experimental deviation caused by the sample preparation can be almost avoided. Secondly, Micro-Raman characterization is repeated on different PAPE samples, and the slight intensity reduction of Raman lines in the low wavenumber range is reproducible. Thirdly, some PAPE experiments of the y-cut samples are repeated to test the reproducibility of micro-crack generation. We find the similar micro-crack topography created by the PAPE treatment. Although the Micro-FT-IR mapping is carried out only once on a series of samples, the result revealed by the mapping data is reproducible: no matter micro-cracks or ablation is induced on the y-cut surface, the anisotropy of the proton distribution along the c-axis is always observed. Nevertheless, for the further quantitative and theoretical study about the PAPE process, more experiments are definitely necessary.

## Conclusion

Impact of the crystal orientation (+c- or y-surface) of LN:Fe on photo-assisted proton exchange and chemical etching is studied in this paper. The proton incorporation is successfully confined in specific regions by incident laser beams with circular and triangle profiles. The intensity reduction of Raman lines in the range of 100–300 cm^−1^ is attributed to the proton incorporation into the Li sites. The +c- and y-surfaces have the different resistance to the PAPE process. The surface morphology of the PACE region on the y-surface shows micro-cracks and lamellar etching fragments along the c-axis of LN. The proton spatial distribution in these cases is elongated along the c-axis. These apparent directional features are attributed to the accumulation of photovoltaic charges at the two sides of the illumination area.
